# Diversity and Impact of Prokaryotic Toxins on Aquatic Environments: A Review

**DOI:** 10.3390/toxins2102359

**Published:** 2010-10-18

**Authors:** Elisabete Valério, Sandra Chaves, Rogério Tenreiro

**Affiliations:** 1 Centro de Recursos Microbiológicos (CREM), Faculdade de Ciências e Tecnologia, Universidade Nova de Lisboa, Quinta da Torre, 2829-516 Caparica, Portugal; Email: evalerio@fct.unl.pt; 2 Centro de Biodiversidade, Genómica Integrativa e Funcional (BioFIG), Faculdade de Ciências, Universidade de Lisboa, Edificio ICAT, Campus da FCUL, Campo Grande, 1740-016 Lisboa, Portugal; Email: sichaves@fc.ul.pt

**Keywords:** diversity of toxins, impact of toxins, prokaryotes, aquatic, molecular mechanisms

## Abstract

Microorganisms are ubiquitous in all habitats and are recognized by their metabolic versatility and ability to produce many bioactive compounds, including toxins. Some of the most common toxins present in water are produced by several cyanobacterial species. As a result, their blooms create major threats to animal and human health, tourism, recreation and aquaculture. Quite a few cyanobacterial toxins have been described, including hepatotoxins, neurotoxins, cytotoxins and dermatotoxins. These toxins are secondary metabolites, presenting a vast diversity of structures and variants. Most of cyanobacterial secondary metabolites are peptides or have peptidic substructures and are assumed to be synthesized by non-ribosomal peptide synthesis (NRPS), involving peptide synthetases, or NRPS/PKS, involving peptide synthetases and polyketide synthases hybrid pathways. Besides cyanobacteria, other bacteria associated with aquatic environments are recognized as significant toxin producers, representing important issues in food safety, public health, and human and animal well being. *Vibrio* species are one of the most representative groups of aquatic toxin producers, commonly associated with seafood-born infections. Some enterotoxins and hemolysins have been identified as fundamental for *V. cholerae* and *V. vulnificus* pathogenesis, but there is evidence for the existence of other potential toxins. *Campylobacter* spp. and *Escherichia coli* are also water contaminants and are able to produce important toxins after infecting their hosts. Other bacteria associated with aquatic environments are emerging as toxin producers, namely *Legionella pneumophila* and *Aeromonas hydrophila*, described as responsible for the synthesis of several exotoxins, enterotoxins and cytotoxins. Furthermore, several *Clostridium* species can produce potent neurotoxins. Although not considered aquatic microorganisms, they are ubiquitous in the environment and can easily contaminate drinking and irrigation water. *Clostridium* members are also spore-forming bacteria and can persist in hostile environmental conditions for long periods of time, contributing to their hazard grade. Similarly, *Pseudomonas* species are widespread in the environment. Since *P. aeruginosa* is an emergent opportunistic pathogen, its toxins may represent new hazards for humans and animals. This review presents an overview of the diversity of toxins produced by prokaryotic microorganisms associated with aquatic habitats and their impact on environment, life and health of humans and other animals. Moreover, important issues like the availability of these toxins in the environment, contamination sources and pathways, genes involved in their biosynthesis and molecular mechanisms of some representative toxins are also discussed.

## 1. Introduction

Toxins are any poisonous substance produced by a living organism that is capable of causing disease or death in other organisms. In several cases, the same organism can produce more than one toxin at the same time. These active products can be considered as part of survival strategies of the producers, as they constitute competitive advantages in the environment. It is not always straightforward to understand the benefit conferred by a certain toxin to a microorganism, but this can be mostly attributed to our limited knowledge about many ecologic, functional and evolutionary aspects of toxin-producing species. In fact, toxins can be considered evolutionary advantages, as they contribute to the survival and/or dominance of a particular organism in a particular environment.

Microorganisms are recognized for their metabolic versatility and ability to produce diverse bioactive compounds such as hydrolytic enzymes, antibiotics, antitumorals and also toxins. Toxins can be produced by prokaryotes such as bacteria [1], in particular cyanobacteria [2], but also by eukaryotes such as dinoflagellates [3], diatoms [3], fungi (mycotoxins) [4,5], and animals (zootoxins or venoms) [1]. With such a broad range of producers, it is expected that toxins present high diversity in chemical composition and mode of action.

The ubiquity of microorganisms in the environment makes them important causes of water and foodborne intoxications, representing central issues in food safety, public health and human and animal well-being. Many of these microorganisms may be present in drinking water supplies or recreational waters. Moreover, the toxins have also great economical impact due to their deleterious effects. Due to their importance, this review will focus on toxins produced by prokaryotic microorganisms in aquatic environments.

Toxins can have diverse natures, including small molecules, peptides, cyclic peptides, lipopeptides, alkaloids, carbamate alkaloids, organophosphates and proteins. Several hundreds of them are known and some have variants with different levels of toxicity.

Toxins present different modes of action and cellular targets, resulting from the chemical nature of the toxin and from their interaction with the target cell. Classification of toxins is not a consensual subject: clinicians often arrange them by the organ they affect (hepatotoxin, neurotoxins, *etc.*), cell biologists prefer to group them according to their effect in the cell (mutagens or carcinogens) and biochemists refer to toxins by chemical origin (e.g., amino acids, peptides, lactones, *etc*). Other possible classification schemes can be defined based on the toxin mode of action, which may be considered a more wide-ranging criterion. Thus, a brief overview of their diversity will be presented based on the type of action that toxins play in the cell.

*Membrane permeabilization:* These toxins start binding to the membrane in their monomeric form. Afterwards, self-oligomerization occurs resulting in the formation of pores that are permeable to ions and small metabolites. Ultimately, this leads to impaired membrane permeability, membrane disruption or osmotic lysis of the cell [6].

*Toxins affecting membrane traffic:* Some toxins can interfere with several components of vesicle-associated membrane protein system, altering the traffic of molecules like neurotransmitters across the membrane (e.g., botulinum toxin) [7,8].

*Toxins affecting signal transduction:* There are toxins that target the intestinal epithelial cells where, after a complex series of events, they activate adenylate cyclase, interfering with signal transduction (e.g., cholera toxin) [9]. Other natural toxins act by modifying key functions of the phosphorylation-based signaling machinery, thus affecting the signal transduction pathways (e.g., microcystins) [2]. 

*Toxins affecting protein synthesis:* This group of toxins can present more than one mechanism to inhibit protein synthesis. Two examples are the cleavage of several nucleobases from the 28S rRNA (e.g., Shiga toxins) [10] or the inactivation of elongation factor 2 (eEF-2) by transferring the adenosine diphosphate ribose moiety (ADP-ribose) of NAD to eEF-2 (e.g., *Pseudomonas* exotoxin A) [11].

*Cytoskeleton-affecting toxins:* These toxins can induce structural changes in the cytoskeleton and consequently inhibit its functions. Cytoskeleton modifications include the disaggregation of actin microfilaments (e.g., Toxin B from *Clostridium dificille*) [12] or the induction of the formation of giant multinucleated cells, leading to changes in actin and tubulin organization (e.g., cytotoxic necrotizing factor of *Escherichia coli*) [13]. 

*Voltage-gated ions channels blockers:* These toxins have the ability to interact with the specific receptors associated with neurotransmitter receptors, or with voltage-sensitive ion channels, therefore inhibiting the nervous signaling (e.g., saxitoxin, kalkitoxin and jamaicamides) [3,14]. 

## 2. Toxins Produced by Cyanobacteria

Some of the most common toxins present in water are produced by cyanobacterial strains of several species. Cyanobacteria represent one of the major bacterial phyla, being an ancient group of prokaryotic microorganisms exhibiting the general characteristics of gram-negative bacteria whose fossil registers date to 3.5 billion years [15,16]. Cyanobacteria constitute an extraordinarily diverse group of prokaryotes. Due to their particular features, they have successfully colonized a wide range of habitats such as fresh, brackish and marine waters, nonacidic hot springs, hypersaline environments, Antarctic soils, rocks, ice and deserts [17,18,19,20]. Only pH seems to restrict the distribution of cyanobacteria, since they tend to prefer neutral or basic conditions and are less common at low pH [19]. They are unique among the prokaryotes, as they have the ability of performing oxygenic photosynthesis, being presumably the first oxygen-evolving photosynthetic organisms during the Precambrian era. They are thought to be also responsible for the transition of the atmosphere of the Earth from its primordial anaerobic state to the current aerobic condition [20].

### 2.1. Blooms and toxicity

Cyanobacterial cell numbers in water bodies vary seasonally as a consequence of changes in water temperature and irradiance, as well as meteorological conditions and nutrient supply. Interactions among phytoplankton organisms in freshwater ecosystems have been detected through changes in the relative abundance of microalgae populations within the phytoplankton communities. In temperate regions, seasonal successions of organisms belonging to different phytoplankton taxa are often observed. Whereas at the beginning of summer a great variety of microalgae and cyanobacteria usually co-exist in the same water body, towards the end of summer this diversity may drop drastically as the result of the massive development of cyanobacterial communities (blooms). One the most known phenomena are the dense blooms of *Trichodesmium erythraeum* that produce a red discoloration of the water and gave the Red Sea its name [19]. Detrimental effects of such cyanobacterial blooms and toxin production are of major concern for water managers. They have become a worldwide increasing problem in aquatic habitats (lakes, rivers, estuaries, and oceans) and in man-made water storage reservoirs. These occurrences can be partially attributed to the gradual eutrophication of the waterways, exposure to constant sunshine, warmth and availability of nutrients like phosphates and nitrates [21]. For example, a low ratio between nitrogen and phosphorous concentrations is one important factor that seems to favor the development of cyanobacterial blooms [18,22]. Since cyanobacteria possess maximum growth rates at temperatures higher than those of green algae and diatoms, the cyanobacterial blooms in temperate water bodies occur mostly during summer months [21,22]. However, there is an unpredictable nature in cyanobacterial blooms and the underlying factors that trigger these phenomena are still poorly understood. As a consequence, the erratic behavior of blooms, in respect to their occurrence, composition, intensity and persistency, demands careful attention in assessing risks for animal and human health.

### 2.2. Importance and impact of the cyanotoxins occurring in aquatic environments

One of the habitats where microorganisms are highly abundant is water. Since many of these microorganisms can produce toxins, they may have a great impact in several living organisms. Toxins produced by cyanobacteria and other microorganisms in sea, rivers, lakes and reservoirs can create adverse effects worldwide. These impact on health and wellbeing, because they are able to induce illness or even death. However, the toxins have also great economic impact due to their deleterious effects.

**Figure 1:**
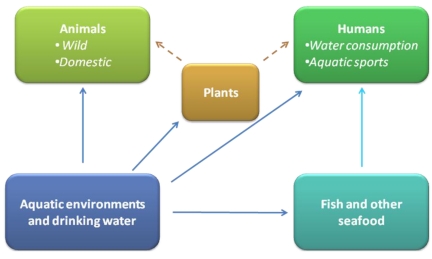
Routes for animal and human intoxication with cyanobacterial toxins.

Contrarily to several other waterborne microbial and toxicant health hazards, which are undetectable to the human eye, cyanobacteria are often readily apparent to the human eye and sometimes olfaction. This is due to the water discoloration, formation of blooms and production of smelling compounds. Cyanobacterial toxic blooms create major threats to animal and human health, tourism, recreation and aquaculture (Figure 1). The occurrence of toxic mass populations appears to have a global distribution [2,23]. The first documented case of a lethal livestock intoxication occurred after consumption of water from a lake heavily populated with cyanobacteria. This was reported in a lake of the Murray River estuary (Australia) by Francis in the 1800s [24]. Nowadays, incidents including both human and animal intoxications have been reported around the world. Lethal animal cases include death of sheep, cattle, horses, pigs, dogs, fish, rodents, amphibians, waterfowl, bats, flamingos, zebras and rhinoceroses [23,24,25,26,27,28,29,30,31,32].

Aquatic recreational activities involving direct contact with contaminated water such as swimming, sailboarding, canoeing and paddling may lead to ingestion, aspiration/inhalation or skin contact with toxic cyanobacterial cells and/or with their toxins. There have been reports about the effects on exposed humans, including respiratory irritation, eye inflammation and severe contact dermatitis. The severity of these effects depends on the toxin dose exposure [23,33,34].

Although most reports of human intoxication caused by cyanobacteria are due to direct ingestion of contaminated water [35,36], chronic intoxication may occur by ingestion of food containing cyanobacterial toxins. For instance, it is known that several cyanobacterial toxins can be accumulated and transferred through the food chain. Microcystins can accumulate in mussels [37,38,39], fish and crustaceans [40], crayfish [41] and even plants that are irrigated with contaminated water [39,42]. Paralytic shellfish poisons (PSP) toxins can accumulate in cladoceran *Daphnia magna* [43], clams, crabs [39] and freshwater mussels [44,45]. Cylindrospermopsin accumulates in mussels [46]. However, there is no evidence of human intoxication risk via bioaccumulation in cattle fed, in neither milk nor beef [47].

So far, numerous bioactive metabolites produced by cyanobacteria have been described, including non-ribosomal peptides, lipopeptides, alkaloids and polyketides that present a vast diversity of structures and variants. Some of them are potent toxins [23,48,49,50,51]. The majority of these peptides are assumed to be synthesized by NRPS (non-ribosomal peptide synthesis, involving peptide synthetases) or NRPS/PKS hybrid pathways, involving peptide synthetases (PS) and polyketide synthases (PKS) [52]. Non-ribosomal peptide synthetases (NRPSs) are multimodular enzymes, found in fungi, cyanobacteria and other bacteria, which biosynthesize peptides without the aid of ribosomes. This kind of biosynthesis allows reaching structures not possible to be obtained by ribosomal peptide synthesis. Most of the non-ribosomal peptides from microorganisms are classified as secondary metabolites, meaning that they do not have a role in primary metabolism, growth or reproduction, but have evolved to somehow benefit the organism that produces it.

Since 2000, major efforts have been made to disclose the genetic basis of the biosynthesis of the compounds produced by cyanobacteria, some of them with unique structures. Here we overview the main gene clusters responsible for cyanotoxins biosynthesis; and discuss similarities and differences among them.

Usually the toxins produced by cyanobacteria are classified according to the effect that they provoke in mammals and vertebrates, where hepatotoxins (liver damaging), cytotoxins (cell damaging), neurotoxins (nerve damaging) and toxins responsible for allergenic reactions (dermatotoxins) have been isolated and characterized from several cyanobacteria [48]. A single species may contain toxic and non-toxic strains; therefore identification at the species level by microscopic morphology does not indicate the potential for toxin production of a given strain. Toxic variations, between and within species of cyanobacteria, are well known from laboratory studies based on isolated cultured strains [53,54,55,56]. So far, an organism able to produce all the variants of each type of toxins or all the types of cyanotoxins has not been described. However, there are some reports on *Cylindrospermopsis raciborskii* strains able to produce several toxins such as cylindrospermopsin, PSPs and unknown compounds [57,58], and also some cases of *Microcystis* strains able to produce microcystins and/or anatoxin-a [59].

### 2.3. Hepatotoxins

#### 2.3.1. Microcystins

One of the most abundant types of cyanotoxins worldwide are microcystins (MC) and are consequently the more intensely studied. An increased incidence of primary liver cancer in China has been associated with the chronic ingestion of sublethal doses of microcystins in raw drinking water [23,60]. The direct uptake of water contaminated with these cyanotoxins through renal dialysis also resulted in some human deaths [36,61].

Microcystins are cyclic peptides with a molecular mass ranging from 900–1100 Da. They share a common structure constituted by Adda-D-Glu-Mdha-D-Ala-L-X-D-MeAsp-L-Z, where X and Z are variable L-amino acids, Adda is the unusual C20 amino acid (3-amino-9-methoxy-2,6,8-trimethyl-10-phenyl-4,6-decadienoic acid), D-MeAsp is 3-methylaspartic acid, and Mdha is *N*-methyl-dehydroalanine [2]. About 80 different variants of microcystins have been described [23,62], with different levels of toxicity. The most common microcystins are MC-LR, MC-RR and MC-YR having the L-amino acids leucine (L), arginine (R) or tyrosine (Y), respectively, in the X position. MC-LR is the most studied variant because of its ubiquity, abundance and toxicity. 

MC are known to be produced by several cyanobacterial genera including *Microcystis*, *Planktothrix*, *Oscillatoria*, *Anabaena*, *Anabaenopsis*, *Nostoc*, *Hapalosiphon*, *Snowella* and *Woronichinia* [18,23,27,34].

The main target of MC is the hepatocyte, the most common cell type in the liver. MC inhibit eukaryotic protein phosphatases and also activate the enzyme phosphorylase b, which results in an excessive phosphorylation of cytoskeletal filaments triggering apoptosis [63]. Death of hepatocytes leads to the destruction of the finer blood vessels of the liver and to massive hepatic bleeding. Some *in vivo* and *in vitro* studies show that organs like kidney and colon can also be affect by the exposure of humans to these toxins [64]. 

**Figure 2:**
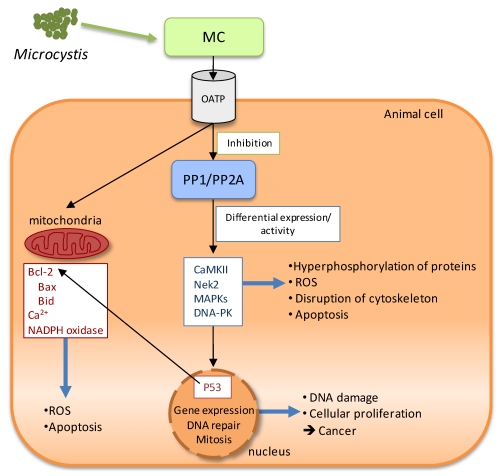
Schematic representation of the molecular mechanisms of microcystins (MC) toxicity. After intake through the plasma membrane by the organic anion transporting polypeptide system (OATP), MC binds specifically to the serine/threonine protein phosphatases (PP1/PP2A), inhibiting them and leading to a cascade of events responsible for the MC cytotoxic and genotoxic effects in animal cells (see text for details).

MC cannot diffuse through the plasma membrane because of their high molecular weight and structure. However, cell specificity and organotropism of MC-LR suggested that a selective pathway for MC uptake would probably exist. Several studies point to the intake of MC through the plasma membrane by a member of the organic anion transporting polypeptide superfamily (OATP) (Figure 2) [64].

Concerning the molecular mechanism of MC toxicity, it is a multi-pathway process, in which the inhibition of serine/threonine protein phosphatases type 1 and type 2A (PP1/PP2A) leads to a cascade of events responsible for the MC cytotoxic and genotoxic effects in animal cells (Figure 2). The mechanisms of tumor promotion are unclear, but apparently they are related to protein phosphatase inhibition leading to hyperphosphorylation of many cellular proteins and deregulation of cell-cycle control. Cell-cycle progression is largely controlled by reversible phosphorylation of regulatory enzymes on their serine/threonine residues. Accordingly, it has been proposed that microcystin induces an increase of oxidative stress, leading to a raise of reactive oxygen species, which can cause DNA damage and is associated with microcystin-induced liver carcinogenesis. In fact, *in vitro* and *in vivo* studies have found oxidative DNA damage in the form of 8-oxo-7,8 dihydro 2’-deoxyguanosine associated with microcystin exposure [65]. 

Through the inhibition of PP1 and PP2A, MC seems to control several cellular processes, e.g., activation of the calcium-calmodulin-dependent multifunctional protein kinase II (CaMKII) by inhibiting its dephosphorylation. The activation of CaMKII may further regulate downstream events such as ROS formation and phosphorylation of proteins including myosin light chain [66]. MC-LR can also activate Nek2 kinase by binding to Nek2 kinase complex with PP1 holoenzyme [67]. Nek2 kinase is a member of the NIMA-related serine/threonine kinase family that participates in the control of mitotic progression and chromosome segregation. This interaction may have implications in the cell viability, tissue injury and tumor development [64]. Moreover, mitogen-activated protein kinases (MAPKs) are serine/threonine-specific protein kinases that regulate several cellular activities, such as proto-oncogenes expression, mitosis, differentiation, proliferation, and cell survival/apoptosis. PP2A mediates MAPKs expression. Therefore the presence of MC probably regulates MAPKs expression as well [68].

MC genotoxicity is also associated with its ability to inhibit two DNA repair systems: nucleotide excision repair (NER) and DNA double strand break (DSB) repair by the nonhomologous end joining (NHEJ). Both systems are regulated by phosphorylation and the inhibition of PP1/PP2A significantly decreases their activity. Furthermore, the inhibition of the DSB-NHEJ pathway is a consequence of loss of activity of the DNA-dependent protein kinase (DNA-PK) resultant from its phosphorylation, after the inhibition of PP2A like enzymes [64]. Additionally, an increase in serine phosphorylation of the nuclear phosphoprotein P53 was identified following both lethal and sublethal MC-LR exposure in mice [69]. This protein is a substrate of PP2A and plays a role as a transcriptional *trans*-activator in DNA repair, apoptosis and tumor suppression pathways [64]. P53 is also a regulator of the expression of the anti and proapoptotic genes including members of the Bcl-2 family such as Bax and Bid. Bax and Bid, play important roles in apoptosis, especially in mitochondria-dependent pathway. Studies indicate that MC-LR can induce mitochondria-dependent apoptosis via the regulation of Bcl-2 family members [70] (Figure 2).

The role of ROS and related mechanisms in MC-LR-induced liver injury *in vivo* are not completely understood. Two possible pathways are mentioned; one of them concerns the outer-membrane permeabilization of the mitochondria after a MC induced massive Ca^2+^ influx, thereby triggering the process of apoptosis. Another plausible mechanism for ROS generation is the increase of NADPH oxidase activity [64] (Figure 2).

Besides what is here presented, there is still much to be done to completely unveil the molecular mechanisms of MC toxicity. 

**Figure 3:**
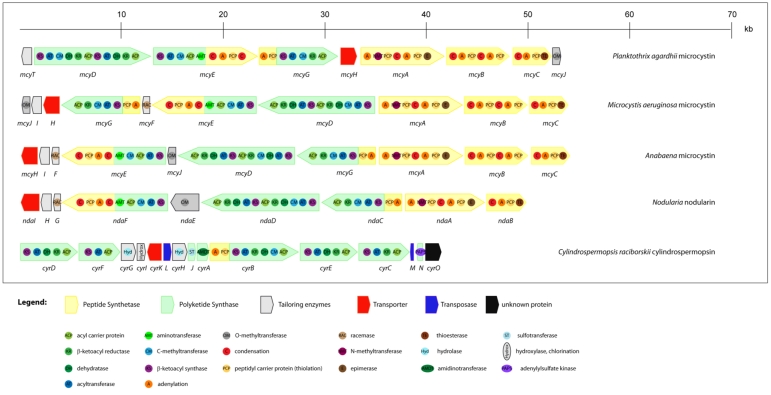
Schematic representation of theorganization of cyanobacterial gene clusters responsible for the biosynthesis of hepatotoxins (microcystins, nodularin, cylindrospermopsin). Different ORFs are indicated as arrows and domains integrated within proteins as circles.

Microcystins are produced non-ribosomally via a thio-template mechanism, by a multienzyme complex consisting of peptide synthetases (PS), polyketide synthases (PKS) and tailoring enzymes. The gene cluster for microcystin biosynthesis was the first to be completely sequenced from a cyanobacterium. It contains approximately 55 kb and is one of the largest bacterial gene clusters described so far. This cluster has been identified and sequenced in three phylogenetic distantly related strains, *Microcystis aeruginosa* PCC 7806 [71], *Planktothrix agardhii* CYA 126 [72] and *Anabaena* sp. strain 90 [73]. Its schematic representation is displayed in Figure 3. This gene cluster consists of nine (*Planktothrix*) or ten (*Microcystis* and *Anabaena*) open reading frames (ORFs). In each module there are specific domains for activation (aminoacyl adenylation domain) and thioesterification (peptide carrier domain) of the amino acid substrate and for elongation (condensation domain) of the growing peptide that is being assembled [71]. The organization of the genes clearly differs among genera. In *Microcystis* and *Anabaena*, the genes are transcribed from a central bidirectional promoter region, whereas in *Planktothrix* all *mcy* genes except *mcyT* seem to be transcribed unidirectionally from a promoter located upstream of gene *mcyD* [74]. However, the multienzyme components are highly similar in the different genera. Except for the tailoring enzymes *mcyI*, *mcyF* and *mcyT*, all other genes *mcyABCDEGH* are always present. However, only the *mcyA-C* arrangement appears to be fairly conserved among toxic strains of the different genera. The *mcyH* gene is an ABC-transporter-like gene and it is thought to be involved in the transport of microcystin [75]. It is assumed that this transporter may be responsible for the localization of the toxin in thylakoids [76,77] or for its extrusion under certain growth conditions [78].

#### 2.3.2. Nodularin

Nodularin is a pentapeptide with a molecular mass of 824 Da. Comparison with microcystin shows the presence of *N*-methyl-dehydrobutyrine (Mdhb) instead of Mdha, and the lack of D-Ala and X residues. So far, this toxin has only been found in *Nodularia spumigena* [2].

Like microcystins, nodularin is a potent tumor promoter that may also act as a carcinogen/tumor initiator and inhibits serine/threonine protein phosphatase-1 and 2A. However, it does not covalently bind to PP1 or PP2A [79]. Due to its structural similarity with microcystins, nodularin is expected to present molecular mechanisms of toxicity similar to those of MC (Figure 2).

A *mcy* homologous gene cluster (*nda*) described in *Nodularia spumigena* NSOR10 is considered responsible for the synthesis of the pentapeptide nodularin [80]. The 48 kb region of the genome consists of nine ORFs (*ndaA-I*) as depicted in Figure 3. Functional assignment of the enzymes was based on bioinformatic analysis and homology to microcystin synthetase enzymes. The *nda* cluster also encodes several putative monofunctional enzymes that may have a role in the modification (NdaE and NdaG) and transport (NdaI) of nodularin.

Studies that have been conducted on the detection and regulation of the genes that are involved in hepatotoxins production are beyond the present scope of this review. The interested reader should see recent reviews [74,81].

### 2.4. Cytotoxins: cylindrospermopsin

Cylindrospermopsin (CYN) is also the object of several studies due its impact, namely in Australia. It is a potent alkaloid, with a molecular mass of 415 Da, consisting of a tricyclic guanidine moiety combined with hydroxymethyluracil. In contrast to MC, the structural variability is much lower. So far, only three variants of the cylindrospermopsin molecule have been described, including deoxy-cylindrospermopsin and 7-epi-cylindrospermopsin, with CYN being more toxic than deoxy-cylindrospermopsin. The presence of guanidino and sulfate groups makes CYN a zwitterionic molecule and hence more soluble in water. Moreover, being a small compound, it is likely to be taken by the cells through diffusion.

CYN and its analogues are known to be produced by some cyanobacterial species, namely, *Cylindrospermopsis raciborskii* [82,83,84], *Umezakia natans* [85], *Aphanizomenon ovalisporum* [86], *Raphidiopsis curvata* [87], *Anabaena bergii* [88], and more recently *Aphanizomenon flos-aquae* [89] and *Lyngbya wollei* [90].

Terao *et al*. [91] described the liver as the main target of this cyanotoxin but other histopathological studies showed that kidneys, thymus and heart are also affected [92,93]. The first clinical symptoms of CYN ingestion are kidney and liver failure [34]. 

**Figure 4:**
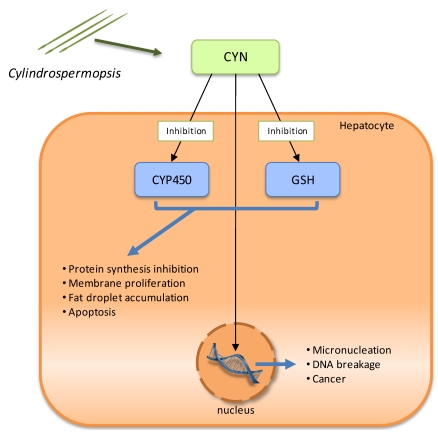
Schematic representation of the known molecular mechanisms involved in Cylindrospermopsin (CYN) toxicity. There is inhibition of glutathione (GSH) and protein synthesis, as well as cytochrome P450 (CYP450), and CYN interaction with DNA.

CYN toxicity results in four pathological changes in the liver: protein synthesis inhibition, membrane proliferation, fat droplet accumulation, and cell death. Despite extensive research, the specific molecular interactions that result in CYN-mediated toxicity are currently unknown. However, it is recognized that this toxin is genotoxic, hepatotoxic *in vivo* and is also a general cytotoxin that blocks protein synthesis. Its toxicity is due to the inhibition of glutathione (GSH) and protein synthesis as well as the inhibition of cytochrome P450 (CYP450) (Figure 4) [94]. There is also evidence of its carcinogenic potential in mice [95]. GSH seems to be required to inactivate cylindrospermopsin, but in the presence of CYN, GSH synthesis is inhibited in the hepatocytes [96].

CYN can also covalently bind to DNA and there is evidence that CYN causes DNA breakage [97]. Therefore, mutagenic activity of the toxin can also be expected. Nevertheless, the exact mode by which CYN causes DNA damage has yet to be determined.

The disclosure of the genes responsible for the biosynthesis of cylindrospermopsin began with Schembri *et al*. [88]. They showed a direct link between the presence of polyketide synthases (PKS) and peptide synthetases (PS) genes in *C. raciborskii* isolates and the ability of those isolates to produce cylindrospermopsin. Later on, Shalev-Alon *et al*. [98] identified amidinotransferase genes (*AoaA*, *AoaB*, and *AoaC*) in an *Aphanizomenon ovalisporum* strain that could be implicated in the cylindrospermopsin synthesis. Recently, using adaptor-mediated gene walking technology, a polyketide biosynthetic pathway, thought to be responsible for the production of cylindrospermopsin, has been described in *C. raciborskii* [94,99]. This cluster spans 43 kb and most of the allocated genes are of PKS nature (Figure 3). However, the biochemical proof for the role of this gene cluster in cylindrospermopsin biosynthesis is still lacking, mostly due to the absence of suitable tools for genetic transformation of *Cylindrospermopsis*.

### 2.5. Neurotoxins

#### 2.5.1. Anatoxin-a and homoanatoxin-a

Anatoxin-a and homoanatoxin-a are unusual alkaloids, secondary amines, with low molecular masses (165 and 179 Da, respectively) exclusively produced by cyanobacteria.

Anatoxin-a is synthesized by various members of the genera *Anabaena* [92], *Cylindrospermum* [100], *Microcystis* [59], *Oscillatoria* [100], *Raphidiopsis* [101], *Planktothix* [102] and *Aphanizomenon* [103]. Homoanatoxin-a is produced by species of the genera *Oscillatoria* [104], *Anabaena* [105], *Raphidiopsis* [101] and *Phormidium* [32]. Some strains are able to produce simultaneously anatoxin-a and homoanatoxin-a [101,106]. 

**Figure 5:**
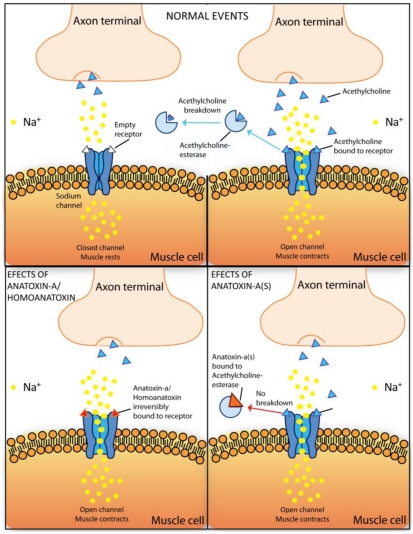
Schematic representation of Anatoxin-a/Homoanatoxin-a and Anatoxin-a(s) molecular mechanisms of toxicity. During “normal events” acetylcholine is released from the neurons, binds to the acetylcholine-receptors on the postsynaptic muscle cell thereby inducing the influx of Na^+^ into the cell. Acetylcholine is degraded by the enzyme acetylcholinesterase in the synaptic cleft into acetate, which is eliminated, and cholin, which is taken up into the neuron by specific carriers. However, in the presence of anatoxin-a and homoanatoxin-a, these toxins bind irreversibly to the nicotinic acetylcholine receptor causing sodium channel opening and the constant inflow of sodium ions to cells. Anatoxin-a(s) causes an irreversible inhibition of the acetylcholinesterase preventing degradation of acetylcholine. The muscles become constantly stimulated.

Anatoxin-a, also previously known as “Very Fast Death Factor”, acts as a post-synaptic neuromuscular blocking agent. Anatoxin-a and homoanatoxin-a are potent agonists of the muscular and neuronal nicotinic acetylcholine receptor. The toxin irreversible binding to the nicotinic acetylcholine receptor causes sodium channel opening and a constant inflow of sodium ions to cells (Figure 5). Overstimulation of the muscle cells occurs as a result of membrane depolarization and desensitization. When respiratory muscles are affected, the lack of oxygen in the brain may lead to convulsions and finally to death of animals by acute asphyxia [47].

**Figure 6:**
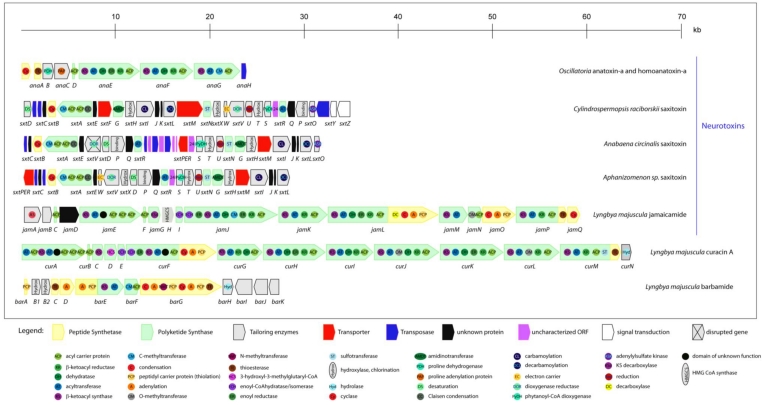
Schematic representation of theorganization of cyanobacterial gene clusters responsible for the biosynthesis of neurotoxins (anatoxin-a and homoanatoxin-a, saxitoxin, jamaicamide), curacin A and barbamide. Different ORFs are indicated as arrows and domains integrated within proteins as circles.

In 2009, the efforts of Cadel-Six *et al.* and Méjean *et al.* showed evidence linking the presence of a 29 kb DNA fragment containing polyketide synthases and the production of anatoxin-a and homoanatoxin-a (Figure 6) [107,108]. The sequence of the gene cluster, assumed to be involved in the production of these toxins, was also obtained by adaptor-mediated gene walking technology. The function of the several identified ORFs was deduced by comparison with other annotated genes and, to date, attempts to genetically confirm the role of this gene cluster in the biosynthesis of these toxins by specific gene disruption have been unsuccessful.

#### 2.5.2. Anatoxin-a(s)

Anatoxin-a(s) is a unique organophosphate with a molecular mass of 252 Da. It is synthesized by *Anabaena flos-aquae* [109] and *Anabaena lemmermanni* [29].

This toxin causes an irreversible inhibition of acetylcholinesterase, which consequently cannot perform the degradation of acetylcholine that is bound to the acetylcholine-receptor [109]. As a result, muscles become constantly stimulated [110] (Figure 5). Its functional consequences are comparable to the one of organophosphorous and carbamate insecticides like paraoxon, physostigmine, pyridostigmine [111] and the chemical warfare agent sarin [112].

#### 2.5.3. Saxitoxin

Saxitoxins (STX), commonly known has paralytic shellfish poisons (PSPs), are tricyclic perhydropurine alkaloids that have a molecular mass of 299 Da. They can be non-sulfated (saxitoxins and neosaxitoxin), single sulfated (gonyautoxins) or doubly sulfated (*C*-toxins) and the possible substitutions at various positions of the molecule results in more than 30 structural variants [2,113,114]. The toxicity of the STX derivatives is different and depends on the type of variant produced; saxitoxin (STX), neosaxitoxin (NEO), and gonyautoxins (GTX1-4) are the most toxic molecules.

Saxitoxins are produced by marine dinoflagellates and cyanobacteria. Members of the freshwater cyanobacteria genera *Anabaena*, *Aphanizomenon*, *Cylindrospermopsis*, *Lyngbya* and *Planktothrix* are able to produce these kinds of toxins [14]. 

**Figure 7:**
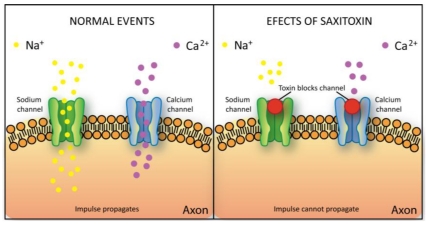
Schematic representation of saxitoxin toxicity mechanism. Saxitoxin binds to the sodium or calcium channels of the nerve axon membranes, preventing the passage of these ions through the cell membrane thus blocking the transfer of the nerve impulse.

Saxitoxin binds to the sodium and calcium channels of the nerve axon membranes, preventing the passage of these ions through the cell membrane and therefore blocking the transfer of the nerve impulse [115,116] (Figure 7). It also extends the gating of potassium channels in heart cells [117]. This action results in a disturbance in the propagation of action potential to muscle cells. Depending on the dose, saxitoxin poisoning may cause symptoms such as tingling and numbness around the lips or, in extreme situations, neuromuscular paralysis and death caused by respiratory failure. It has also been shown to exhibit a cardio depressant effect [118].

Saxitoxins are among the most toxic compounds known. Nowadays, saxitoxins are already included in the Schedule 1 of the Chemical Weapons Convention together with warfare agents such as mustard gas, sarin, ricin and others [118,119].

In 2008, Kellman *et al*. revealed the biosynthetic pathway of STX production using reverse genetics to identify the candidate STX biosynthetic gene cluster (*sxt*) in the cyanobacterium *Cylindrospermopsis raciborskii* T3 [120]. Functional assignment of the enzymes was based on bioinformatic analysis combined with the liquid chromatography-tandem mass spectrometry analysis of the biosynthetic intermediates; however, the biochemical proof for the role of this gene cluster in saxitoxin biosynthesis is lacking, because *C. raciborskii* is not genetically transformable. In *C. raciborskii*, the *sxt* gene cluster spans approximately 35 kb and presents genes responsible for toxin biosynthesis, regulation and export (Figure 6). However, some of the identified genes have not yet been assigned to a function due to their low level of homology with proteins present in databases.

Later on, in order to obtain the sequences of the gene cluster also responsible for SXT biosynthesis in *Anabaena* and *Aphanizomenon*, a gene walking technique (pan-handle PCR) was employed by Mihali *et al.* [114]. It revealed a 29 kb gene cluster in *Anabaena circinalis*, and a slightly smaller cluster in *Aphanizomenon* of 27.5 kb (Figure 6). The bioinformatically-deduced functions reveal that the cluster presents some variations between the genera, namely in the genes assumed to be involved in toxin regulation, and there are also some differences regarding the genes supposedly involved in toxin transport.

### 2.6. Lipopeptides from marine cyanobacteria

Marine cyanobacteria are amazing in the diversity of new biologically active natural products synthesized using mixed NRPS/PKS systems. Several lipopeptides have been purified from the marine cyanobacteria *Lyngbya majuscula*. For example, Kalkitoxin and jamaicamides A, B, and C are neurotoxins that block voltage-gated sodium channels, while antillatoxins A and B activate them [14]. Metabolites with pharmacological importance, like barbamide (used in the biological control of snails), the anticancer compound curacin A and antifungal agents as hectochlorin, have also been identified.

#### 2.6.1. Jamaicamides

Jamaicamide A is a highly functionalized lipopeptide containing an alkynyl bromide, vinyl chloride, β-methoxy eneone system, and pyrrolinone ring. Jamaicamide B is a debromo analogue of jamaicamide A, while in jamaicamide C, which also lacks the bromine atom, a terminal olefin replaces the terminal alkyne of jamaicamide B. Jamaicamides A, B and C have all been isolated from *Lyngbya majuscula*.

Jamaicamides have been demonstrated to present a sodium channel blocking activity (Figure 7) and fish toxicity.

The biosynthetic pathway responsible for jamaicamides synthesis has been recently investigated [121]. Based on feeding precursor experiments to jamaicamide-producing cultures, an effective cloning strategy for the biosynthetic gene cluster discovery was developed. The 58 kb gene cluster has 17 open reading frames (Figure 6), the majority of PKS nature, showing a notable co-linear arrangement with respect to its proposed utilization during biosynthesis [121]. 

#### 2.6.2. Kalkitoxin

Kalkitoxin is a thiazoline-containing lipid derivative also produced by the pantropical marine cyanobacterium *Lyngbya majuscula*. 

It has been indirectly shown that kalkitoxin blocks voltage-gated sodium channels (Figure 7) [122]. Furthermore, this toxin has proven to be ichthyotoxic to the goldfish *Carassius auratus* and toxic to the crustacean brine shrimp *Artemia salina* [123].

#### 2.6.3. Antillatoxins

An extremely potent ichthyotoxic *L. majuscula* metabolite, antillatoxin A, was firstly reported in 1995 [124]. Antillatoxin is a structurally remarkable lipopeptide, presenting a high degree of methylation. It is among the most ichthyotoxic metabolites isolated, and is only exceeded in potency by the brevetoxins [124].

The studies performed so far to determine its mechanism of action showed that it activates the mammalian voltage-gated sodium channel at a pharmacological site that is distinct from any previously described [125]. Antillatoxin B is a variant of antillatoxin A, which has reduced sodium channel-activation properties and exhibits less ichthyotoxic activity [126].

#### 2.6.4. Curacin A

Curacin A is a unique natural product presenting a structure with two lipid chains and sequential thiazoline and cyclopropyl rings.

Curacin is promising as an antiproliferative agent due to its inhibitory action on tubulin polymerization. Since it has been shown to block cell cycle progression by interacting with the colchicines binding site on tubulin and inhibiting microtubule polymerization, this compound may have value in the treatment of neoplasic disorders [127].

The genetic basis involved in curacin production has been described by Chang *et al*. [128]. A combined approach employing isotope incorporation and molecular genetics was employed to reveal the biosynthetic pathway of curacin. The bioinformatic analysis showed that this gene cluster spans 63.7 kb, containing 14 ORFs (Figure 6), making this the largest gene cluster described in cyanobacteria. The genetic architecture of the cluster shows a co-linear arrangement with respect to its expected utilization during biosynthesis. This amazing cluster is almost only composed by PKS modules, with the exception of *curF*, which is a hybrid PKS/NRPS bimodule.

#### 2.6.5. Barbamide

Barbamide is a chlorinated lipopeptide that has been isolated due to its molluscicidal activity [129].

The biosynthetic pathway responsible for this toxin production has started to be revealed based on incorporation studies using isotope-labeled precursors. In 2002, Chang *et al.* have described the complete sequence of this unusual gene cluster containing NRPS and PKS modules [130]. Sequence comparison with databases showed the existence of 12 putative ORFs allocated in the 26 kb cluster (Figure 6). However, clear evidence of the involvement of each ORF of this gene cluster in barbamide biosynthesis will only be achieved by performing heterologous expression or gene disruption assays.

In this review, the diversity of the NRPS or NRPS/PKS hybrid pathways, involving peptide synthetases and polyketide synthases used by cyanobacteria to produce toxins with unusual structures (Figure 3 and Figure 6), is shown. Most of the toxins (microcystins, nodularin, cylindrospermopsin, jamaicamide, curacin A and barbamide) have two main modular biosynthetic systems: (i) the non-ribosomal peptide synthetases (NRPSs), responsible for assembling amino acids leading to peptide formation, and (ii) the polyketide synthases (PKSs), used to link together acetate as the primary building block. Since NRPS and PKS enzymes are able to accept a wide range of different substrates, a huge number of possible different structures can be reached using these systems.

In NRPS, a minimal elongation module, that is responsible for one elongation step, comprises three catalytic domains: an adenylation domain (A), responsible for substrate recognition and activation by adenylation; a thiolation domain (T), needed for the covalent incorporation as thioesters; and one condensation (C) domain, for condensation of the precursor [131] (e.g., *P. agardhii mcyB* gene in Figure 3). Epimerization (E) or *N*-methylation (NMT) domains may also be present in the module, leading to further substrate modification (*P. agardhii mcyA* gene in Figure 3).

The cyanobacterial PKS consists of multiple sets of domains and modules, which normally correspond to the number of acyl units in the product [132]. One module comprises a set of domains that are responsible for the activation, modification and elongation of a single amino acid or carbon unit. A minimal multifunctional module is composed of a ketoacyl synthase (KS) domain, an acyltransferase (AT) domain and an acyl carrier protein (ACP) domain (e.g., the *cyrF* gene of *C. raciborskii* cylindrospermopsin cluster). Frequently, ketoreductase (KR), dehydratase (DH) and enoyl reductase (ER) domains are constituents of megasynthases, as the *anaE* gene of *Oscillatoria* anatoxin-a and homoanatoxin-a cluster.

In the case of the neurotoxins clusters, namely saxitoxin, an extraordinary number of secondary tailoring manipulations, including oxidation, methylation and diverse forms of halogenations are also present.

With the exception of the *Lyngbya majuscula* clusters, all the other described clusters include ORFs responsible for the toxin transport (red arrows in Figure 3 and Figure 6). This evidence indicates that the release of the cyanobacterial toxins to water does not necessary implies cell lysis, since some toxins may be actively exported from the cells.

Another interesting feature of cylindrospermopsin, anatoxin-a/homoanatoxin-a and saxitoxin gene clusters is the presence of ORFs correspondent to transposases, which can lead to speculation that the presence of these transposases may have an important role in the horizontal transfer of these genes.

### 2.7. Lipopolysaccharides

Endotoxic lipopolysaccharides (LPS) are part of the outer membrane of gram-negative bacteria, including cyanobacteria. LPS and its effects are well known from bacteria such as *Escherichia coli*, *Salmonella* spp., *Vibrio cholera*, *Yersina pestis* and *Pseudomonas aeruginosa*. LPS composition includes lipid A, core polysaccharides and an outer polysaccharide chain. Opposing to the other bacteria, LPS from cyanobacteria have a higher diversity of long chain unsaturated fatty acids, hydroxyl fatty acids and lack phosphate [39].

It is accepted that LPS cause fever in mammals and are involved in septic shock syndrome [133]. Besides their action on the immune system, LPS from bacteria and cyanobacterial origin also affect the detoxication system of different organisms [39]. However, LPS from bacterial origins have shown to be more toxic than the cyanobacterial ones.

## 3. Toxins Produced by Other Bacteria in Aquatic Environments

Although cyanobacteria are the most important group of aquatic toxigenic prokaryotes, there are other bacteria, present in aquatic environments, which can produce toxins with high relevance for human and animal health. *Vibrio* spp. are one of the most representative groups of aquatic toxin producers, commonly associated with seafood-born infections and intoxications. *Aeromonas hydrophila* is also associated with aquatic habitats and has been described as responsible for the production of toxins [134]. Furthermore, recognized pathogens like *Escherichia coli*, *Campylobacter* spp. or *Legionella pneumophila* are also water contaminants and have been described as emergent toxin producers. An overview about several aspects of these water associated toxin producers will be presented, including the diversity of toxins, their impact on environment and human life, molecular mechanisms, cellular consequences, pathways and genes involved in their biosynthesis. 

The diversity of bacterial toxins is high and more potentially toxic molecules are emerging. In this review we will focus on some representative examples, chosen based on the specificity of their molecular mechanisms and their impact on human and animal life.

### 3.1. *Vibrio* spp.

*Vibrio* species belong to Gamma-proteobacteria, are curved usually motile rods, mesophilic and presenting a chemoorganotrophic or facultative fermentative metabolism [135]. They are highly abundant in aquatic environments, including estuaries, marine coastal waters and aquaculture facilities [136,137,138,139]. They also appear to be highly associated with marine organisms like fish [140,141], mollusks [142,143] and shrimps [144,145], which are important food products for human consumption. *Vibrio* organisms present another important feature: they can attach to the exoskeletons of crustaceans and other marine organisms of the zooplankton, producing biofilms [137]. Their close relationship with zooplankton can be a survival strategy to resist to environmental stresses like starvation or antibiotic presence [146]. This strong association to zooplankton is of utmost importance, as they can easily enter into the human and animal food webs. On the other hand, some *Vibrio* species are recognized as relevant pathogens for animals reared in aquaculture [147,148]. Fish and shellfish mortality caused by vibrios is very frequent in early larval stages [149,150]. Sometimes *Vibrio* spp. infections can lead to the death of entire populations, with high economical consequences. Three *Vibrio* species (*Vibrio cholerae*, *Vibrio parahaemolyticus* and *Vibrio vulnificus*) are considered serious human pathogens. Both *V. cholerae* and *V. vulnificus* produce toxins that are fundamental as virulence factors. Some of the most relevant toxins produced by these species will be addressed in more detail.

#### 3.1.1. *Vibrio cholerae*

This species is the causative agent of cholera, a severe disease that had a central role in the history of infectious diseases. Cholera outbreaks are reported since 1817. Presently, Cholera continues to be responsible for thousands of deaths, especially in developing countries, where poor water supply and poor sanitation are unsolved problems [151,152]. *V. cholerae* is found in coastal, estuarine and marine environments, often associated with aquatic fauna such as copepods and shellfish and is transmitted to humans by contaminated water and food [151,152]. Close relationships with zooplankton are also established and cholera outbreaks have been associated with planktonic blooms and sea surface temperatures. The wide ecological relationships of *V. cholerae*, the ability to form biofilms and to adapt to environmental changes have highlighted the pathogenic potential of this species.

The main virulence factor associated to *V. cholerae* pathogenesis is the production of the potent cholera toxin (CT). Cholera is characterized by a voluminous watery diarrhea, leading to rapid dehydration. Patients can lose as much as 20 liters of fluid in 24 hours and more than 50% of them die if not treated. The clinical aspects of the disease are primarily induced by the activity of this toxin [151], but not all strains are able to produce it. In fact, more than 200 *V. cholerae* serotypes have been described, but only two (O1 and O139) can produce the CT-toxin [151,153].

The genes for toxin synthesis (*ctx*AB) are carried by the lysogenic bacteriophage CTXΦ and only strains with the integrated phage are able to produce CT-toxin [154]. The expression of these genes is coordinated with the expression of other virulence factors like TCP (toxin-coregulated pilus, coded by *tcp* genes and required for intestinal colonization) and the accessory colonization factors (coded by the *acj*A–D genes). Virulence genes are located in a pathogenicity island of *V. cholera* genome [151]. The primary direct activator of virulence genes transcription (including *ctx*ABC) is ToxT protein. It belongs to a large protein family (AraC/XylS) that shares a domain of 100 amino-acids, which corresponds to a helix-turn-helix DNA binding motif and has transcription activation functions [155]. AraC domain is nearly invariant among all ToxT sequences, but a second domain (NTD) was identified in these proteins, being much less conserved. Its function is not clear, but it is thought that this domain is the binding site for a natural effector, which is proposed to be bile[156,157]. ToxT activates transcription by binding to a degenerate 13-bp DNA sequence (the toxboxes), located upstream of all genes activated by this protein. These toxboxes can occur in different configurations at different promoters and can be organized as direct or inverted repeats, never overlapping the -35 promoter element [158]. The activity of ToxT in *ctx*AB promoter is mainly to counteract the H-NS histone-like protein that binds to the same region, but strongly represses the *ctx*AB gene expression. ToxT also interacts directly with the α subunit of RNA polymerase, activating transcription [159]. The regulatory pathways of CT toxin are complex, involving upstream regulation of *toxT* expression (regulatory proteins as ToxR, TcpP and ToxS have already been identified and characterized), interaction with effectors and coordination with the expression of other virulence genes. The regulatory pathways of CT production and expression of other virulence factors were recently reviewed by Matson *et al.* [151]. 

After entry in the intestinal lumen, *V. cholera* interacts with intestinal microenvironment, sensing a luminal factor that induces a low expression level of TCP and enables the adherence of the bacteria to the intestinal mucosa, probably to the glycocalyx. A second environmental signal, dependent on microbial adherence to the mucosa, is detected and enhances the level of TCP expression, promoting the intestinal colonization and finally inducing the production and secretion of CT toxin [160,161]. ToxR and TcpP, both inner membrane proteins, are critical for these events but the nature of the signals that activate them remain unclear. 

CT-toxin is a bipartite molecule belonging to the AB_5_ family, which also includes Shiga and pertussis toxins. CT combines one A active subunit and five identical peptides (~11 kDa) that is assembled into a highly stable pentameric ring named B subunit (~55 kDa). The A subunit presents two domains: A_1_ and A_2_. These two peptides are linked by an exposed loop containing a protease-sensitive “nick” site and a single disulfide bond. 

The B subunit has a high affinity to the oligosaccharide domain of the G_M1_ ganglioside on the surface of the intestinal epithelial cells, allowing the binding of the toxin to the plasma membrane of host cells. B pentamer binds stoichiometrically to five G_M1_ gangliosides at cell surface [161]. G_M1_ functions to concentrate CT in glycolipid-rich apical microdomains (“lipid rafts”) located in the cell surface [162]. These lipid rafts are distinct cholesterol rich membrane structures that act as membrane organizing centers for signal transduction, protein and lipid sorting, endocytosis and transcytosis. The raft structure and role depends on the specific lipids that compose the microdomain and the specific binding of the toxin to GM_1_ gangliosides depends on these lipids. This step is considered to be the critical step for the subsequent targeting of the toxin into all intracellular compartments, required to trigger the cellular response. A stable binding between B subunit and G_M1_-receptor complexes is essential for CT function. 

Considering the A subunit, the A_2_ peptide (~5 kDa) attaches the A_1_ peptide (~22 kDa) to the B subunit and presents a COOH-terminal KDEL motif that extends from B pentamer on the side that binds G_M1_ [163]. This KDEL motif is known to be a sorting signal that allows endogenous proteins from endoplasmic reticulum (ER) to be retrieved efficiently from post-ER compartments. A_1_ is the enzymatic active subunit of the toxin and must dissociate from B subunit and translocate across a cellular membrane to act on its cellular target.

**Figure 8:**
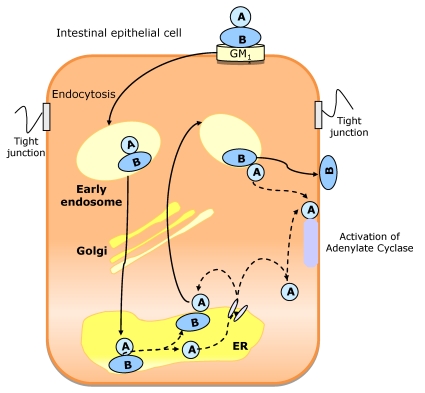
Proposed mechanism of cholera toxin (CT) traffic into intestinal epithelial cells. The CT holotoxin binds to GM_1_ in the apical membrane. After endocytosis, the CT-GM_1_ complex traffics retrograde through Golgi cisterna into endoplasmic reticulum (ER). Here, the A_1 _subunit is unfolded and dissociated from the B pentamer. The unfolded A_1_ peptide is probably translocated to the cytosol and may then gain access to its substrate, the heterotrimeric GTPase Gsα on the cytoplasmic surface of the basolateral membrane, by diffusion through the cytosol (if the A1 peptide breaks away from the membrane after translocation) or by membrane traffic back out of the secretory pathway (if the A1 peptide remains membrane associated). The B subunit is not unfolded in the ER, remaining membrane associated, probably bound to GM_1_. It moves to the basolateral membrane by trafficking back out the secretory pathway by indirect transcytosis.

CT is not a pore forming toxin. Rather it uses the host cell membrane traffic machinery, entering into the intestinal cell through a complex mechanism. The proposed pathway (Figure 8) starts with the binding of CT holotoxin to G_M1_ in the apical membrane. The G_M1_-CT complex enters the cell by apical endocytosis and traffics retrograde through Golgi cisternae into the ER lumen. Then A_1_ peptide unfolds, is translocated to the cytosol, and breaks away from the membrane after translocation. Then it can move by diffusion to its cellular target (adenylate cyclase complex), located on the cytoplasmatic surface of the basolateral membrane. Alternatively, the active subunit of the toxin can remain membrane associated. In this case, it moves back out of secretory pathway into the cytosol by vesicular transport back [162]. The B subunit does not translocate across cell membranes, remaining membrane associated (probably bound to G_M1_ receptors) and moving back out of the secretory pathway by vesicular traffic to the cell surface. This mechanism allows B subunit to move from its original binding site (apical/mucosal) to the basolateral site (basolateral/serosal) through Golgi cisternae and ER. 

The cellular target of CT is the adenylate cyclase complex at the basolateral membrane. The active subunit A_1_ acts as an enzyme that is able to specifically transfer ADP-ribose group to an arginine residue of the α subunit of the GTP-binding protein Gs. Once activated, this Gs α subunit dissociates from the Gs membrane-bound subunit and crosses the cell to attach to the catalytic unit of adenylate cyclase in the basolateral membrane. Once activated, this enzyme induces the formation of cAMP and the subsequent activation of cAMP-dependent protein kinase. Finally, the phosphorylation of membrane proteins involved in the transepithelial ion transfer induces changes in the ion transport. The final consequences of this process are the inhibition of Na^+^ and Cl^−^ absorption in villous cells and the stimulation of secretion of Cl^−^, HCO_3_^−^ and water in epithelial cells resulting in massive electrolyte loss and dehydration [151,153,164].

Strains not carrying *ctx* genes can also be pathogenic, as they have several virulence factors and can produce other toxins like RTX cytotoxins [165], which will be discuss further in the context of toxins produced by *V. vulnificus*. Another *V. cholera* toxic metabolite is the cholix toxin [153]. The existence of the cholix toxin was discovered by detailed analysis of non-01 and non-139 strain genomes that revealed the presence of a gene encoding a putative new exotoxin, similar to ExoA from *Pseudomonas aeruginosa* [166]. 

Similarly to ExoA toxin from *P. aeruginosa*, cholix toxin is recognized by the lipoprotein receptor-related protein (LRP) of the host cells, enabling access to the cell cytoplasm. However, some recent studies performed in LPR receptor-deficient cells point to the existence of other paths for toxin entry, as these cells showed some sensitivity to the cholix molecule. Cholix toxin presents a KDEL sequence, responsible to direct it to ER, and is activated within the host cell by furin cleavage in an Arg-rich loop, together with the reduction of critical disulfide bridges [153]. The toxin is an ADP-ribosylating protein that is specific to ribosomal eEF-2 elongation factor. It recognizes eEF-2 as the target protein substrate and has both glycohydrolase and ADP-ribosylating activities that are necessary to change diphthamide, the post-translationally modified histidine residue of this eukaryotic elongation factor. The modification mechanism involves the transfer of the ADP-ribose complex of NAD^+^ to the diphthamide imidazole in eEF-2 via a nucleophilic substitution were diphthamide imidazole is the nucleophile that replaces the nicotinamide base (leaving group) in NAD^+^. Exactly how ADP-ribosylation of the diphthamide inhibits eEF-2 function remains to be determined. Binding experiments of ADP-ribosylated eEF-2 to the ribosome show a reduction of affinity for the pre-translocational ribosome, but no changes are observed for the post-translocational ribosome. Competition and co-sedimentation experiments have indicated that the ADP-ribosylated eEF-2 is able to form a stable complex with the ribosome. However, other binding experiments have shown that ADP-ribosylated eEF-2 still has ribosome-dependent GTPase activity and can dissociate from the ribosome. These contradictory results confirm that further studies are necessary to obtain a better understanding of how these ribosylating toxins inactivates the elongation factor [166]. Nevertheless, independently from the mechanism of eEF-2 inactivation, the final consequence for the host cell is the inhibition of protein synthesis and cell death.

This toxic protein is coded by the *chx*A gene, corresponding to a 666 amino acid residue product (634 residues in the mature protein). The first 32 residues correspond to a leader sequence. It presents a sequence identity of 33.5% to ExoA (the catalytic domains alone have a sequence identity of 41.3%). In addition, *chx*A contains conserved residues previously shown to be crucial for catalytic activity [166]. Full length structure of the protein demonstrated that cholix toxin is composed by three different domains (I–III) that are responsible by receptor binding, membrane translocation and enzyme catalysis, respectively [153].

After diphtheria toxin and ExoA from *P. aeruginosa*, the *V. cholerae* cholix toxin is the third member of this group of protein synthesis inhibitors described so far. The *chx*A gene is widely distributed in *V. cholerae* populations, both in clinical and environmental strains. However, the specific targets of the toxin and the symbiotic interactions associated with its activity have yet to be determined.

#### 3.1.2. *Vibrio vulnificus*

This *Vibrio* species is part of the natural microbiota of coastal marine environments and is frequently present in water and seafood products like shrimp, fish, oysters and clams [167,168,169]. It is an opportunistic human pathogen, responsible for severe and fulminant systemic infections that are highly lethal [170]. Consumption of seafood, especially raw oysters, is the main human contamination source. The characteristics of *V. vulnificus* infections include fever, chills, nausea, hypotensive septic shock and secondary lesion formation on the extremities of the body [171]. Primary septicemia is the most lethal infection, with about 50% mortality rate. In addition, this bacterium can cause serious wound infections as a result of exposure to contaminated waters. Wounds are often acquired during recreational swimming, fishing or seafood handling [172].

Two cytotoxins (an elastolytic protease, VvpE, and a hemolysin, VvhA) were first suggested to be responsible for the toxic effects of *V. vulnificus* [173,174]. However double mutants for the corresponding genes did not present significant differences in cytotoxicity from the wild type strain [175]. Later it was shown that *V. vulnificus* strains are cytotoxic due the production of a repeats-in-toxin (RTX) exoprotein [176]. This toxin is also present in several bacterial species including V. *cholerae*, and was first detected in this species, but is the main toxin in *V. vulnificus*, being essential for its virulence and infection. 

RTX toxins, including the RtxA1 from *V. vulnificus*, primarily promote pore formation in cellular membranes, but can also induce changes in cytoskeletal structure, bleb formation and aggregation of actin, resulting in cell rounding. In *V. vulnificus* infection, actin aggregation occurs without covalent cross-linking and is a depolymerization process that involves a change in the F/G actin dynamics for which Rho GTPases play important roles. These changes can lead to cellular necrosis and are probably related with the bacteria’s ability to destroy gastrointestinal epithelial/mucosal barrier and invade blood stream soon after infection [176]. RTX operon contains its own processing and ABC transporter genes. After production RtxA1 is autoprocessed in at least two parts (approximately 350 and 130 kDa) and this activation mechanism is induced by the contact with host factors [176]. Only the processed protein can act as cytotoxic factor.

RTX proteins are usually large molecules, presenting GD-rich nonapeptide repeats (GGXGXDX[L/I/V/W/Y/F]X, where X is any amino acid). This structure is thought to be involved in the insertion of the toxin in the eukaryotic plasma membrane [165]. However, the toxin of *V. vulnificus* and *V. cholerae* has some particular characteristics. The repeats are different, falling into three classes, presenting divergent sequences, but retaining a central conserved motif (G-7X-GXXN). The class A motive is also located in the *N*-terminus of the protein and not in the *C*-terminal part. Furthermore, the presence of the multifunctional autoprocessing domain in these toxins shows that they are part of a particular group of RTX toxins, called multifunctional autoprocessing repeats-in-toxins (MARTX). These toxins present several structural features that are different from other RTX toxins [165]. The RtxA1 from *V. vulnificus* is estimated to be the largest RTX toxin known so far. The predicted amino acid sequence showed high homology with RtxA from *V. cholerae*. However, two domains show no identity with RtxA and it was hypothesized that these two regions may confer distinct activities to these toxins. RtxA1 toxin is likely to be much more cytotoxic to the host cells than RtxA from *V. cholerae*. These two domains may play an important role in pore formation in host cell membrane, which can be related with the cytotoxic mechanisms of RtxA1 [176]. 

Identification of this toxin was possible by random chromosomal mutagenesis that allowed the detection of the corresponding gene (*rtx*A1). Two additional *rtxA* genes have also been identified, but their function is not clear, as they are not directly involved in cytotoxic activity. The first portion of the *rtx* operon (*rtxBDE*) includes genes for the type I secretion system that is responsible for the toxin secretion [177]. The second part of the operon (*rtxAC*) includes the *rtxC* gene that codes for an activator of RtxA in *V. cholerae*, but probably has other function in *V. vulnificus*, as a mutation in this gene does not affect toxicity. Other potential regulator of *rtxA* (HlyU) was also identified in *V. vulnificus*. It is a transcriptional regulator that binds upstream of the *rtx* operon, initiating transcription [178]. 

### 3.2. *Aeromonas hydrophila*

*Aeromonas* spp. are members of Aeromonadaceae that cause both intestinal and systemic infections in humans. *Aeromonas hydrophila* colonizes aquatic environments and is also isolated from food products [179]. Although gastroenteritis occurs generally in young children, it has been frequently associated with the travel’s diarrhea [180]. Furthermore, the cases of septicemia are often fatal. This species can express several virulence factors, including hemolysins, proteases, adhesins, lipases/phospholipases and toxins [181]. This bacterium also has the ability to lyse erythrocytes, and has shown to be invasive and effective in triggering the proinflammatory response in macrophages and epithelial cell lines [179,182]. Act is an aerolisyn-related pore-forming toxin that is responsible for the hemolytic, cytotoxic and enterotoxic activities of *A. hydrophila*, being its main virulence factor. 

Hemolysis involves pore formation in the membrane of the target cell and water entry from the external media, resulting in swelling of the cells and subsequent lysis. The toxin interacts with the membranes of erythrocytes, inserts into the lipid bilayer as oligomers, and creates pores in the range of 1.14 to 2.8 nm. Cholesterol serves as the receptor for Act and the 3’-OH group of this membrane constituent is important for the interaction. Once Act has interacted with cholesterol on the cell membranes, the toxin is activated with subsequent oligomerization and pore formation [179]. 

**Figure 9:**
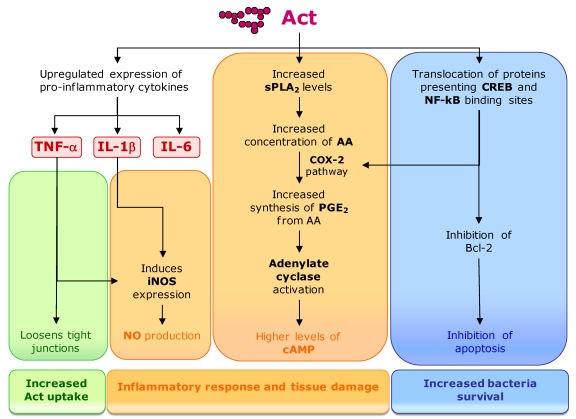
Act mediated pathways for activation of inflammatory response and apoptosis inhibition (see text for details).

The toxin activity also includes tissue damage and high fluid secretion in intestinal epithelial cells, resulting from the induction of a proinflammatory response in the target cells (Figure 9). Act upregulates the production of proinflammatory cytokines such as tumor necrosis factor alpha (TNF-α), interleukin-1 beta (IL-1β) and IL-6 in macrophages. TNF-α and IL-1β stimulate the production of the inducible nitric oxide synthase (iNOS) that, through nitric oxide (NO) production, is an essential element of antimicrobial immunity and host-induced tissue damage. Simultaneously, Act has the ability to activate arachidonic acid (AA) metabolism in macrophages that leads to the production of eicosanoids (e.g., prostaglandin E2 [PGE_2_]) coupled to cyclooxygenase-2 (COX-2) pathway. AA is a substrate for PGE2 production, but is present at limited concentrations in cells [182]. Act increases the amount of AA from phospholipids by inducing group V secretory phospholipase A_2_ (sPLA_2_), which acts in the membrane of eukaryotic cells. Act increases cyclic AMP (cAMP) production in macrophages by indirect activation of adenylate cyclase by PGE_2_. The *A. hydrophyla* toxin also induces the production of antiapoptotic protein Bcl-2 in macrophages, preventing the occurrence of massive apoptosis resulting from the induction of the inflammatory response, which would be undesirable for the bacteria. Act also promotes an increased translocation of the nuclear factor kB (NF-kB) and cAMP-responsive element binding protein (CREB) to the nucleus [182]. Transcription factor NF-kB is important in a number of inflammation-related pathways. The enhancer/promoter regions of some immunoregulatory cytokine genes, including the TNF-α, IL-1β, and IL-6, present binding elements for NF-kB and CREB [183]. These transcription factors have also important regulatory functions in the transcription of *cox-2* and are implicated in the induction of Act cytotoxic activities.

The production of proinflammatory cytokines and iNOS causes an extensive tissue injury in the intestinal loops. It also loosens the tight junctions around epithelial cells, allowing the influx of inflammatory cells into the intestinal lumen and increasing the uptake of Act into lamina propria, where inflammatory cells can be activated. Moreover, PGE2 along with cAMP leads to the stimulation of fluid secretory response and the subsequent fluid loss [182]. 

The mature protein is 52 kDa and contains 493 amino acids. It is secreted as an inactive precursor and undergoes processing at both the *N*- and *C*-terminal ends to demonstrate biological activity. It has a leader sequence of 23 amino acids that allows the protein to transverse the inner membrane. This leader peptide is removed when the toxin enters the periplasmic space. After the secretion of Act into the medium, a polypeptide of approximately 4–5 kDa (about 45 amino acids) is cleaved from its *C*-terminus by a protease produced by *A. hydrophila*, resulting in the mature form of the toxin. This toxic protein is coded by *A. hydrophila act* gene, corresponding to a 1479 bp open reading frame. The first 69 bp codes for the *N*-terminal signal peptide. The typical regulatory -35, -10 and Shine-Delgarno sequences, as well as the promoter, were identified upstream of the coding region [179,184,185].

More recently, two other toxins were detected in *A. hydrophila*. Alt is a 44 kDa protein, with 368 amino acid residues, coded by the corresponding *alt* gene. The other is Ast, the product of a 1911 bp open reading frame that originates a protein of 71 kDa and 636 amino acids. Both represent new molecules with no significant homology to other bacterial toxins. Although there is evidence of their contribution to the elevated cAMP and prostaglandin E2 levels in infected cells, their specific roles must be clarified [181].

### 3.3. *Escherichia coli*

*Escherichia coli* is a genetically heterogeneous group of bacteria from the Enterobacteriaceae families, whose members are generally non-pathogenic and are part of the normal microbiota of the intestinal tract of humans and animals [186]. However, some strains have acquired genes that enable them to cause diseases [187]. These strains can be divided into two types (pathotypes), based on the mechanisms and virulence factors by which they cause disease [187]. One of these *E. coli* pathotypes (STEC) [187] corresponds to the enterohemorrhagic strains and is characterized by the production of at least one type of Shiga toxin, a family of structurally and functionally related exotoxins, which includes the toxin produced by *Shigella dysenteriae* [188]. This *E. coli* toxin is also known as verotoxin due to its effect in Vero cells. Ruminants, in particular cattle, constitute a vast source of STEC and is frequent that human infections are originated in food and water contaminated with cattle manure, especially because they are carried by healthy animals [186]. Infections in humans may result in water diarrhea, bloody diarrhea or in the hemolytic uremic syndrome (HUS), characterized by acute renal failure, hemolytic anemia and other severe symptoms. The kidney and the gastrointestinal tract are the most affected organs, but lungs, heart, central nervous system and pancreas can also be targeted. HUS develops in 5–10% of individuals infected with STEC O157:H7, the most frequent serotype causing this infection in humans [188].

Shiga toxins are encoded in the genome of several lambda-like bacteriophages, which represent highly mobile genetic elements and play a central role in horizontal gene transfer [189]. Phage *stx* genes are located in the late region, downstream of the late promoters, and are highly expressed during the lytic cycle [188]. The folding and assemblage of the toxins are only possible in the particular conditions of bacterial periplasm and their release seems to occur by phage mediated bacterial lysis. Two types of Shiga toxin are recognized: Stx1 and Stx2, coded by the corresponding genes *stx1* and *stx2*. Stx1 is highly conserved in structure and similar to Shiga toxin from *S. dysenteriae*. However, a variant Stx1c was already identified and has been mainly associated to ovine origins [190]. In contrast, there are several antigenic variants of Stx2: Stx2c, Stx2d, Stx2d-activatable and Stx2e. Stx2d and Stx2e are believed to be associated to mild or asymptomatic diseases and Stx2c is believed to be less frequent in patients presenting severe symptoms [186].

**Figure 10:**
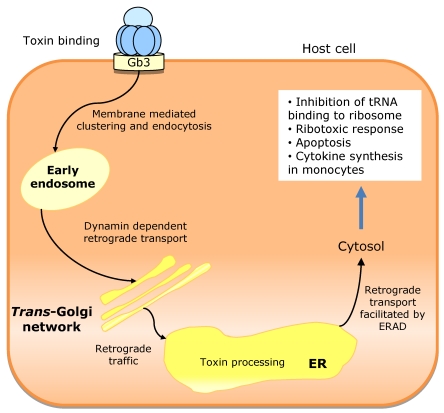
Trafficking mechanism of Shiga toxins. Toxin binding to the plasma membrane induces membrane-mediated clustering and the toxin-driven endocytosis. The toxin then undergoes retrograde sorting in early endosomes, in a dynamin-dependent process. Shiga toxins bypass the late endocytic pathway and are transferred directly from the early endosome to the *trans*-Golgi network (TGN) and from there to the endoplasmic reticulum (ER). Finally, Shiga toxins use the ER-associated degradation (ERAD) machinery to facilitate retrotranslocation into the host cell cytosol. There it can reach its cellular targets.

The Stx molecules (approximately 70 kDa) present an A_1_B_5_ hexameric structure of toxins from AB_5_ family, in which A subunit (32 kDa) is non-covalently linked to the pentamer of B subunits (7.7 kDa each). Subunit A (StxA) is enzymatically active and the B fragments (StxB) are responsible for host cell binding [186,188]. StxB binds to the neutral glycosphingolipid globotriaosylceramide (Gb3), which is present at the surface of susceptible cells, allowing the internalization of the toxin [191]. Each StxB fragment binds to three trisaccharide sites. Sites 1 and 2 mediate high affinity receptor binding and are the most relevant for cell cytotoxicity. The third site mediates the recognition of additional low affinity Gb3 epitopes [192]. Distinct variants of the Stx toxin present some conformational divergences in site 2 and show affinity for Gb3 receptors with different fatty acid chains [193]. After binding to the specific receptor in the plasma membrane, Stx enters the cell by endocytosis. The toxin can induce endocytic invaginations in the plasma membrane without the help of cell machinery. Membrane blending results from the glycosphingolipid receptors aggregation, mediated by Stx [194]. The membrane invaginations are then processed by the cell mechanisms, involving dynamin, actin and membrane cholesterol. After entry into the cell, Shiga toxin localizes in early endosomes, but it escapes the late endocytic pathway and is directly transferred to *trans*-Golgi network (TGN) and then to ER. Dynamin and retromer are molecules implicated in membrane blending and have shown to be important for the direct transfer of Stx from early endosome to TGN [195,196]. The transport mechanism from TGN to ER is unknown, but is independent from the coat protein complex I (COPI). Shiga toxin does not induce pore formation in the cell membrane. Like RtxA from *V. cholerae*, it relies on host cell machinery to translocate the A active subunit to the cytosol. During the early entry process, a protease sensitive loop in the *C*-terminal region of StxA (residues 242–261) is cleaved by a membrane-associated endoprotease (furin), originating two StxA fragments: the catalytic fragment A1 (amino acids 1–251) and the StxB associated fragment A2 (amino acids 252–293). A1 domain remains linked to StxA2-StxB complex by a disulfide bond, which is reduced in ER lumen, releasing the catalytic domain that is subsequently translocated to the cytosol [186,194] (Figure 10).

Shiga toxin has a highly specific RNA-glycosidase activity that cleaves an adenine base from the 28S ribosomal RNA (rRNA) of the eukaryotic ribosome [197]. The 3’ end of 28S rRNA functions in the aminoacyl t-RNA binding, peptidyltransferase activity and ribosomal translation. This modification of ribosomes inhibits the tRNA binding to the ribosome and the subsequent chain elongation. It also triggers a signaling response termed ribotoxic response that includes the activation of the JUN *N*-terminal kinase and p38 (a mitogen-activated protein kinase—MAPK), altering the signaling of the extracellular signal-regulating kinases ERK1 and ERK2 [198]. The toxin also activates several cellular kinases, like tyrosine kinases that phosphorylate several proteins including dynamin, which favors toxin uptake, and p38, implicated in ribotoxic response [199]. Shiga toxin damages the microcirculation and the intestinal mucosa, leading to bleeding into the bowel and bloody diarrhea. This provides essential nutrients to the bacteria, favoring its survival [188].

**Figure 11:**
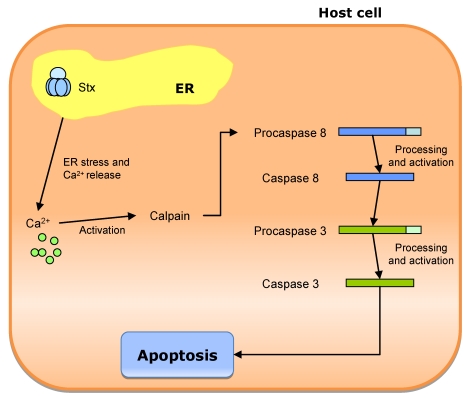
Apoptosis pathway triggered by induction of ER stress response and Ca^2+^ mediated calpain activation.

Shiga also induces cytokine synthesis and release. Some monocytes and macrophages are resistant to the toxin and respond to toxin binding and internalization by producing and releasing pro-inflammatory cytokines, which in turn stimulate the Gb3 biosynthesis and expression in several endothelial cells, promoting the cytotoxic action of the toxin. After crossing the intestinal epithelium and entering in circulatory system, Stx stimulates the monocytes to secrete cytokines like GM-CFS and TNF [200]. Interleukin 8 (IL-8) probably plays a central role in this process. All these reactions can contribute to the endothelial cell damage. Furthermore, Stx can signal apoptosis by a mechanism that requires retrograde transport through Golgi apparatus and ER and the activation of caspase 3. Caspases are cysteine-dependent, aspartate-specific proteases that are a central component of the programmed cell death pathway. Shiga toxin induces the ER stress response, a cellular mechanism that is usually activated under the accumulation of unfolded and misfolded proteins, leading to Ca^2+^ release from ER stores [201,202]. Ca^2+^ activates cysteine protease calpain that activates caspase 8 through cleavage. This last protease directly activates caspase 3, initiating the apoptotic process (Figure 11).

Bacterial ribosomes are also susceptible to Stx toxin [203], decreasing the proliferation of susceptible bacteria from the intestinal microbiota and allowing a more efficient propagation of the pathogen.

### 3.4. *Legionella pneumophila*

*Legionella pneumophila* is the pathogenic organism responsible for the Legionnaires disease, a potential lethal pneumonia that results from the ability of this bacterium to survive and replicate in macrophages. No animal reservoir is known, but its natural hosts and environmental source are aquatic protozoa (e.g., amoebae), in which they replicate and seems to enhance its ability to infect mammalians cells [204]. The human infection occurs mostly by inhalation of aerosols generated by domestic and environmental water sources [205]. *L. pneumophila* cells enter the macrophages by vacuoles that are immediately surrounded by vesicles or mitochondria and move towards the endoplasmic reticulum, escaping from fusion with lysosomes and reaching a perfect niche for their multiplication [206].

*L. pneumophila* also produces a toxin of the RTX family (of which the general structure is discussed above), a pore-forming protein that has an important role in adherence to host membranes and in the molecular traffic of the bacteria during the infection process [207]. Its role in bacterial adherence and traffic may be due to the ability of these proteins to bind β_2_ integrin receptors in the target cell membranes [208]. After entering the cell, this toxin induces pore formation in the vacuoles, preventing proper docking of lysosomes and fusion. This process allows bacterium survival inside the host cell.

In this species, RTX toxin (RtxA) is a large protein (about 7000 amino acids), with a variable number of repeated units and a modular structure. The toxin appears to be clearly divided in two regions: the *N*-terminal, involved in cell adhesion and the *C*-terminal, involved both in adhesion and pore-forming. The repeats at the *N*-terminal end are also highly variable among strains [209]. Analysis of the RtxA genes in several strains showed the existence of long tandem repeats (460–549 bp), variable in number and sequence, and with a high level of rearrangements and diversity when compared with the flanking regions [209]. The corresponding ORFs range between 4669 to 7910 bp. Different kinds of cell adhesion domains were identified in the *N*-terminal region of *rtx* genes of the diverse strains. Two of these motifs were always present: (i) the von Willebrand factor A (VWA) domain, involved in cell membrane adhesion processes, and (ii) other tandem repeat domain with homology with the hemolysin calcium-binding site, which is related with adhesion to other host surfaces and pore formation [209].

Recently a glucosyltransferase from *L. pneumophila* (Lgt1) was identified. This *Legionella* enzyme (a protein with 60 kDa) modifies the eukaryotic eEF-1A. This elongation factor is a GTP-binding protein, possessing GTPase activity. Lgt1 alters the serine-53 of eEF-1A, located in the GTPase domain. The modification results in inhibition of protein synthesis both *in vitro* and *in vivo* and causes extreme changes in cellular morphology and, ultimately, death of intoxicated eukaryotic cells [210]. This bacterium is able to multiply inside phagocytes. Toxin expression is only induced after a successful replication, when bacteria go out to the surrounding medium, being important to the transmission to a new host. 

*L. pneumophila* have several open reading frames encoding unknown proteins that can correspond to unknown toxins and justify further studies on this species.

### 3.5. *Campylobacter* spp.

The genus *Campylobacter* includes curved, S-shaped and spiral rods, with a microaerophilic metabolism and presenting spherical or coccoid cells in old cultures or under oxygen exposure [211]. Two species of this genus (*Campylobacter jejuni* and *Campylobacter coli*) are important causes of diarrheal diseases worldwide. *Campylobacter* spp. infections usually cause severe gastroenteritis, but can also be responsible for prolonged enteritis, bacteremia, septic arthritis and other infections [212]. *Campylobacter* spp. are zoonotic and many animals are natural reservoirs for human disease, including poultry, cows, sheep and pigs. Campylobacters are frequently isolated from water, and water supplies have been a source of some outbreaks [211]. The pathogenic mechanisms and virulence factors responsible for *Campylobacter* spp. gastroenteritis are not completely understood, but like other enteric pathogens, *Campylobacter* spp. colonize, invade and transmigrate across human intestinal cells. The interaction of the bacteria with the intestinal epithelium induces the production of several pro-inflammatory cytokines, including interleukin-8 (IL-8), a major cytokine secreted by the intestinal epithelial cells. IL-8 functions as a chemoattractant, recruiting neutrophils to the site of infection, contributing to the inflammatory response. One virulence factor already identified is a cytolethal distending toxin (CTD) that induces distention of the cytoplasm of infected cell, increase in their DNA content and accumulation of the inactive tyrosine phosphorylated form of Cde2, a key regulator of cell cycle progression [213]. This type of toxin has already been found in other microorganisms like *Shigella* spp. or *Helicobacter hepaticus* [214,215]. This toxin is also implicated in the induction mechanism of IL-8 production [216]. CTD is required for IL-8 production, in addition to toll-like receptors (TLRs), which play a central role in initiating the inflammatory response. *Campylobacter jejuni* cytotoxic strains secrete CTD by a flagellum mediated mechanism and activate NF-kB. The production of this nuclear transcription factor is stimulated both by CTD and TLRs, via MyD88 signaling TLRs adaptor, and triggers IL-8 [216].

CDT is an AB_2_ heterodimeric tripartite toxin, composed of three subunits: CdtA, CdtB and CdtC. Amino acid sequence of the CtdB subunit shows high similarity with members of DNase A nuclease family. Although limited to some residues, they correspond to a motif that is essential for nuclease activity. Thus, CtdB is the active subunit of CTD, acting by DNA damaging and stopping cell cycle in G_2_/M phase [217]. CtdA and CtdC fragments are involved in the delivery of the active subunit CtdB into the cell [218]. The toxin is encoded by three genes, *ctdA*, *ctdB* and *ctdC* that are located in a chromosomal operon.

## 4. Water Contaminating Toxin Producing Bacteria

Other toxigenic species like *Clostridium* spp. and *Pseudomonas* spp. are ubiquitous in the environment. Even though they are not aquatic microorganisms, they can easily contaminate drinking or irrigation waters and become hazards for humans and animals. Some representative examples of toxins produced by these bacteria will be briefly referred below.

### 4.1. *Clostridium* spp.

*Clostridium* is a genus of pleomorphic, gram-positive, anaerobic rods that are widespread in several habitats, including soils, wetlands, lakes, coastal waters, intestinal track of fish and gills and viscera of crabs and other shellfish [219]. They are highly resistant to adverse environmental conditions as they are able to produce endospores. These bacterial spores are cellular structures that can resist to extreme temperatures, desiccation, chemicals and radiation. When favorable growth conditions are reestablished, the spores can germinate, originating viable vegetative cells [135]. Recreation and drinking water can became contaminated with *Clostridium* spp. spores from sources like soils and dust, and insects also contribute for their spread. *Clostridium* spp. cells are not pathogenic by themselves. However several species may produce exotoxins that can be extremely hazardous [219]. 

#### 4.1.1. *Clostridium botulinum*

Botulism, the disease cause by C. *botulinum*, is a severe neurological illness that causes paralysis. It is originated by a potent neurotoxin produced by this bacterium. Seven antigenically distinct botulism toxins (types A, B, C1, D, E, F and G) were identified and are considered among the most toxic substances known. Botulism toxins (BoNTs) are water soluble large molecules (150 kDa), produced as single peptides. The active toxin is composed by a heavy chain (H) and a light chain (L), linked by a disulfide bond. It is originated after proteolytic cleavage by endogenous bacterial proteases [220]. BoNTs may form oligomers, which can be involved in channel formation and translocation of the protein into the host cytoplasm. The toxin can also form complexes with other proteins such as nonhemagglutinin (NTNH) and hemagglutinines [221,222]. These spontaneously formed complexes confer toxin resistance to proteases and their association is pH dependent. Complexes are maintained at pH lower than 7.2 and spontaneously dissociate at higher values. The toxin acts by blocking the neurotransmission at peripheral motor nerve terminals. They selectively hydrolyze proteins involved in the fusion of synaptic vesicles with the presynaptic plasma membrane, preventing acetylcholine release [223]. This process occurs in four steps: binding, membrane translocation, internalization and intracellular action [224]. The H chain is responsible for the selective binding to neurons, internalization of the total toxin and intraneuronal sorting. L chain blocks exocytosis after toxin release in the cytoplasm [225].

The neurotoxin genes are located in a transcriptional unit, together with the genes encoding NTNH and hemagglutinins. This unit is referred as BoNT gene complex [226]. The location of BoTN genes and associated nontoxic proteins depends on the type of toxin. BoNT A, B, E and F genes are located in the bacterial chromosome together with the genes of the corresponding associated proteins [227]. BoNT gene complexes of serotypes C1 and D are coded by a bacteriophage [228]. Genes of serotype G are located in a plasmid [229]. In each gene complex, NTNH gene is located immediately upstream of BoNT genes in all toxin types [226]. Genes for the other components are clustered upstream of NTNH gene for most of the toxins. Only C1 type presents a different arrangement, including three transcriptional units: (i) the first including NTNH and BoTN genes, (ii) the second composed by three genes coding hemagglutinins and (iii) the last one, with only one gene, probably having a regulatory function [228].

#### 4.1.2. *Clostridium perfringens*

The virulence of *Clostridium perfringens* largely results from its ability to produce toxins. At least 14 different toxins have been identified, but each strain only carries genes for a defined subset of the total repertoire. This characteristic provides the basis for a toxin typing system that groups *C. perfringens* isolates into five types (A to E), depending on its ability to produce four (alpha, beta, epsilon and iota) of the 14 toxins [230]. The total set of toxins includes two that are active in the human intestinal tract: *C. perfringens* enterotoxin and beta-toxin, each one associated with a distinct disease. One is necrotic enteritis, caused by type C strains, with the beta-toxin considered as the main virulence factor. This toxin is related to alpha-toxin and leukocidin from *Staphylococcus aureus*. Beta-toxin is considered a single component toxin, presenting a molecular mass of about 35 kDa and 334 amino acids [202]. The first 27 residues correspond to a signal peptide, directing the export of the toxin across the cell membrane. The toxin is encoded by a single open reading frame of approximately 1006 bp (*cpb* gene) with the Shine-Dalgarno sequence located at 7 bp upstream the ATG start codon [231].

*C. perfringens* type A is responsible for most of the human diseases caused by this species. The symptoms of the infection (diarrhea and cramping) are due to the *C. perfringens* enterotoxin (CPE), a peptide presenting a unique amino acid sequence and mechanism of action. CPE is a thermo-labile protein with 319 amino acids, without *N*-terminal secretion sequence and presenting a molecular mass of 35 kDa [232]. The production of this enterotoxin is associated with the sporulation process of *C. perfringens* and can represent more than 10% of the total protein in sporulating cells [232]. The toxin is released when the mother cell lyses at the end of the sporulation. Thus, during this process, much CPE is accumulated in paracrystalline inclusion bodies in the cytoplasm of *C. perfringens* cells [232]. The action of CPE is a multistep process. It starts with the binding of the toxin to its intestinal receptors, inducing the formation of a small complex that entraps CPE at the membrane surface. This complex interacts with other proteins, forming a second complex of intermediate size, which can interact with occludin (a tight junction protein) or other proteins of the eukaryotic cell. This causes the loss of membrane permeability characteristics, probably because the complex has pore-like properties or by inducing tight junction rearrangements [211]. The *N*-terminal end of the toxin has the cytotoxic activity and the *C*-terminus is likely to be involved in binding to cell membrane. The CPE coding gene (*cpe*) can be located either in the bacterial chromosome or in a conjugative plasmid. The corresponding ORFs are identical. The expression of *cpe* is induced by regulators (e.g., alternative sigma factor) that are involved in the sporulation process, explaining its high production in sporulating cultures [232]. One possible regulator is the Hpr global regulator, as Hpr-like binding sequences have been identified upstream and downstream of *cpe* ORF [233]. Since Hpr is known to repress the expression of some genes during exponential growth of *Bacillus subtilis*, the role of Hpr in CPE expression could explain the lack of expression of the toxin in vegetative cells.

### 4.2. *Pseudomonas aeruginosa*

*Pseudomonas* is a genus of gram-negative, rod-shaped and mobile bacteria that demonstrate a great metabolic diversity and ability to use a high number of organic substrates, including phenol derivatives and hydrocarbons [234]. A significant number of species can also produce exopolysaccharides and create biofilms [235]. These two characteristics highly contribute to their ability to colonize a wide range of habitats, being widespread in the environment, including plants, animals, soils and water.

*Pseudomonas aeruginosa* is increasingly recognized as an emerging opportunistic pathogen of clinical relevance [236]. As with other species, it is ubiquitous in the environment and can easily adapt to adverse conditions. The human infections range from acute infections like endocarditis, meningitis and septicemia to chronic lung infections in cystic fibrosis patients. Most infections occur in immunocompromised patients, like AIDS patients, burn victims or patients undergoing chemotherapy [237]. *P. aeruginosa* presents several virulence factors, including the exotoxin A (ExoA/PE). It belongs to the same family of mono-ADP-ribosyltransferases of *V. cholerae* cholix toxin, which catalyses the ADP rybosilation of eukaryotic eEF-2 and consequently inhibits protein synthesis [238]. PE is translated from an ORF with 2760 bp as a monocistronic message and is a protein of 613 amino acids, resulting from a 638 amino acid precursor that includes a hydrophobic leader peptide of 25 amino acids [237]. As described before for cholix toxin, PE presents three distinct functional domains. The first, the *N*-terminal Ia domain (a.a. 1–252) is responsible for cell recognition [239]. Afterwards, the toxin enters the cell and is internalized in early endosomes, where it is cleaved by the protease furin, originating two fragments: a *N*-terminal with 28 kDa and a *C*-terminal with 37 kDa [240]. This last one is the active fragment and is released in a pH dependent process. Then it is transported to the endoplasmatic reticulum via late endosomes and Rab9-dependent route to the *trans*-Golgi network and finally traveling by the KDEL receptor mediator pathway [241,242]. When the enzymatic subunit of PE becomes present in the cytosol, it promotes the ADP ribosylation of the eEF-2. The ADP-ribosylation mechanism develops as previously described for cholix toxin, inactivating eEF-2 and inhibiting protein synthesis. Consequently, it leads to cell death.

## 5. Final Remarks

Bacteria are ubiquitous in the environment and have the ability to adapt to very different habitats. Their survival is often dependent on the production of compounds that help them to attach to substrates, compete for nutrients and inhibit the growth of other microorganisms. Many virulence factors are products that give them advantages in their original environment, but in a host they function as pathogenic mechanisms of disease development. Some of these products represent powerful toxins that can lead to host disease and, frequently, to death. On the other hand, water is not only a crucial resource but is also a requisite to maintain life. The contamination of water systems by toxigenic microorganisms can have a catastrophic impact in health and well being of all living organisms. Toxins or toxin producers in water systems can arise either by contamination from other environmental sources (e.g., *Clostridium* spp. [219], *P. aeruginosa* or *E. coli* [243]) or because aquatic systems are their natural habitats (e.g., cyanobacteria, *Vibrio spp*. [244] or *Aeromonas hydrophila* [245]). Microbial toxins are very diverse and there are many microorganisms recognized as producers. Although this review only focuses on the more relevant toxins and toxin producers, more toxic molecules are emerging in other organisms. An overview of the toxins produced by bacteria related to aquatic environments is summarized in Table 1.

**Figure 12:**
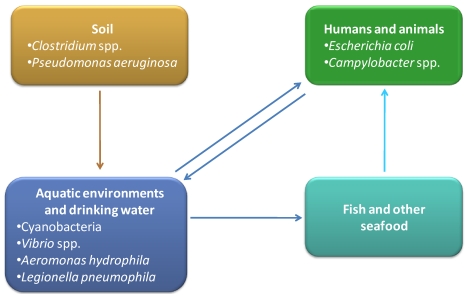
Natural reservoirs of the main bacterial toxin producers and routes for water, animal and human contamination.

In fact, a better knowledge about all aspects of these microorganisms seems crucial. Identification of routes and sources of water contamination (Figure 12) will allow the design of preventive actions to avoid this contamination or to prevent conditions that favor the development of toxic microorganisms and toxin production. 

**Table 1:** Toxins produced by prokaryotes related to aquatic environments.

Mode of action	Toxin name	Produced by	References
Membrane permeabilizing toxins	Act	*A. hydrophila*	[179]
	α-Hemolysin	*E. coli*	[1]
	Bifermentolysin	*C. bifermentans*	[1]
	Botulinolysin	*C. botulinum*	
	Chauveolysin	*C. chauvoei*	
	Histolyticolysin O	*C. hystolyticum*	
	Novyilysin	*C. novyi* A	
	Perfringolysin O	*C. perfringens*	
	Septicolysin O	*C. septicum*	
Toxins affecting membrane traffic	Botulinum neurotoxin	*C. botulinum*	[220]
Toxins affecting signal transduction	Cholera toxin	*V. cholerae*	[151]
	Heat-labile enterotoxin	*E. coli*	[1]
Toxins affecting protein synthesis	Cholix toxin	*V. cholera*	[153]
	Exotoxin A	*P. aeruginosa*	[237]
	Shiga toxin (verotoxin)	*E. coli*	[187]
	Lgt1	*L. pneumophila*	[210]
	RtxA	*V. vulnificus*	[165]
	RtxA	*L. pneumophila*	[207]
Toxins inhibiting protein function	Cylindrospermopsin	*Cyl. raciborskii*	[82,83,84,85,86,87,88,89,90]
		*Umezakia natans*	
		*Aph. ovalisporum*	
		*Raph. curvata*	
		*A. bergii*	
		*Aph. flos-aquae*	
		*Lyngbya wollei*	
	Microcystins	*Microcystis*	[27,51]
		*Planktothrix*	
		*Oscillatoria*	
		*Nostoc*	
		*Anabaena*	
		*Anabaenopsis*	
		*Hapalosiphon*	
		*Snowella*	
		*Woronichinia*	
		*Arthrospira*	
		*Phormidium*	
		*Plectonema*	
		*Pseudoanabaena*	
		*Synechococcus*	
		*Synechocystis*	
	Nodularins	*Nodularia spumigena*	[2]
Cytoskeleton-affecting toxins	Anatoxin-a and homoanatoxin-a	*Anabaena*	[32,59,100,101,102,103,104,105,106]
		*Oscillatoria*	
		*Cylindrospermum*	
		*Microcystis*	
		*Aphanizomenon*	
		*Planktothrix*	
	C2 toxin	*C. botulinum*	[246]
	Cytotoxic necrotizing factors	*E. coli*	[1]
DNA damaging	Cytolethal distending toxin	*Campylobacter* spp.	[213]
Voltage-gated ions channel blockers	Saxitonin and gonyautoxins	*A. circinalis*	[14]
		*Aph. gracile*	
		*C. raciborskii*	
		*L. wollei*	
		*Planktothrix*	
	Jamaicamides	*Lyngbya majuscula*	[14]
	Kalkitoxin		
	Antillatoxin		
Unknown	Lypopolysaccharides (LPS)	*E. coli*	[39,133]
		*Salmonella* spp.	
		*V. cholera*,	
		*P. aeruginosa*	
		Cyanobacteria	

Clarification of the mechanisms of toxin action will certainly open new perspectives for efficient antidote as well as vaccine development [246,247]. Furthermore, the potential of these toxic products as anticancer, antifungal or antibiotic drugs are now recognized and can be an important source for biomedical and biotechnological applications in the near future [188,237,246].
